# A mixed-methods analysis of mobility behavior changes in the COVID-19 era in a rural case study

**DOI:** 10.1186/s12544-021-00472-8

**Published:** 2021-02-10

**Authors:** Alexandra König, Annika Dreßler

**Affiliations:** 1 Institute of Transportation Systems, German Aerospace Center (DLR e.V.), Lilienthalplatz 7, 38108 Braunschweig, Germany; 2 Institute of Transportation Systems, German Aerospace Center (DLR e.V.), Rutherfordstraße 2, 12489 Berlin, Germany

**Keywords:** Travel behavior, COVID-19, Mixed methods, Rural areas, Case study

## Abstract

**Background:**

As a reaction to the novel coronavirus disease (COVID-19), countries around the globe have implemented various measures to reduce the spread of the virus. The transportation sector is particularly affected by the pandemic situation. The current study aims to contribute to the empirical knowledge regarding the effects of the coronavirus situation on the mobility of people by (1) broadening the perspective to the mobility rural area’s residents and (2) providing subjective data concerning the perceived changes of affected persons’ mobility practices, as these two aspects have scarcely been considered in research so far.

**Methods:**

To address these research gaps, a mixed-methods study was conducted that integrates a qualitative telephone interview study (*N* = 15) and a quantitative household survey (*N* = 301). The rural district of *Altmarkkreis Salzwedel* in Northern Germany was chosen as a model region.

**Results:**

The results provide in-depth insights into the changing mobility practices of residents of a rural area during the legal restrictions to stem the spread of the virus. A high share of respondents (62.6%) experienced no changes in their mobility behavior due to the COVID-19 pandemic situation. However, nearly one third of trips were also cancelled overall. A modal shift was observed towards the reduction of trips by car and bus, and an increase of trips by bike. The share of trips by foot was unchanged. The majority of respondents did not predict strong long-term effects of the corona pandemic on their mobility behavior.

## Introduction

As a reaction to the novel SARS-COV-2 virus that was first reported in December 2019, countries around the globe have implemented various measures to reduce the spread of the virus. Especially social distancing measures and stay-at-home mandates were chosen by governments worldwide to interrupt the transmission of the virus by separating infected and uninfected persons [48, 62]. Social distancing, banning of unnecessary travel, the closure of borders, home office measures and the general reduction of reasons for travelling have had immediate impacts on people’s social interactions and mobility behavior [1]. A lot of these measures affected the mobility of people and goods [4, 6]. Human mobility is found to play an important role in the epidemic growth rate of the virus [39]. The transportation sector is particularly affected by the current coronavirus disease situation [29, 61]. Furthermore, public transport use assumed to play a decisive role for the transmission of the virus [55]. Adding on this, data monitoring on human mobility and migration can be used to understand and predict the spread of COVID-19 [55].

### Early empirical findings on changes in mobility behavior due to the measures to control the coronavirus situation

The ongoing global fight against the spread of the coronavirus invited research to collect and analyze data on the effects of the measures on transport systems [47]. Technology companies like Google and Apple that collect and analyze data of movement trends by regions in mobility reports found a worldwide drop in journeys [5, 33]. First empirical insights from traffic statistics and analyses of smartphone positions pointed to an enormous reduction of personal mobility [36, 38, 51, 52]. A study from France revealed that the decrease in mobility was uneven across the time of the day, with movements during rush hours being the most disrupted due to school closure and remote working [52]. The reduction of personal mobility was shown to be caused maily by the reduction of everyday commute [36]. Furthermore, studies reveal a reduction in the number of grocery shopping trips [20, 23].

However, the effect of the pandemic on transport differed by mode. A reduction of personal mobility was particularly shown for public transport trips [2, 4, 13, 20, 36, 38]. In Germany, a survey among more than 2000 individuals revealed that one fourth of the respondents stopped using public transport, while another 17% stated to have reduced trips in public transport [2]. Contrarily, the share of private car trips increased [2, 23]. A considerable share of respondents in a German study expressed their intention to buy a car as a reaction to the COVID-19 situation [23]. This trend towards an increase of the relative share of private car trips within the remaining mobility was shown in further studies from Hungary [13], Spain [4] and China [34]. At the same time, studies reveal an increase in the number of trips by bike [36, 45] and an increase of cycling in the modal share [4, 34].

The existing early research work emphasizes the need for studies that address the long-term effects of measures against the spread of the virus for the transport sector and personal mobility [36]. However, the lack of empirical data and insights concerning the perception and assessments of persons make it difficult to predict the effects of the pandemic situation on mobility behavior in the next years. Empirical studies are needed to describe the disruptive potential of the crisis on peoples ongoing mobility behavior and behavioral adaptations and to identify ways in which transport policy can facilitate adaptations that are desirable to society [41]. There are various approaches to describe and explain mobility behavior and transport mode choice. The paper adapts points of view on mobility behavior based on attitude-based theories, like the Theory of Planned Behavior [3], that take norms, beliefs and attitudes into account for describing mobility behavior. Attitude-based theories of mobility behavior assume individual’s affective reactions to using a system as a predictor of the intention to use it and the actual behavior [58]. With regard to potential changes in mobility behavior as a reaction to the crisis, attitude-based theories propose that besides attitudes, normative beliefs and perceived behavioral control affect the mobility behavior.

Psychological and individual factors, like age and education level, have been shown to be associated with the engagement in protective behaviors during a pandemic [12]. For the current pandemic situation, e.g. a recent study has revealed differences in perceived vulnerability and perceived risk as well as in preventive behaviors, according to age, gender and other demographic characteristics. Women expressed a significantly higher perceived risk and fear of the new coronavirus and showed higher commitment to preventive behaviors, compared to men [64]. Furthermore, first studies suggest an important role of demographic disparities with regard to reactions to the COVID-19 situation [57], as observed, for example, in a higher likelihood of persons with higher socioeconomic status to evade the city [17]. Giving regard to the fact that highly populated areas like megacities reported a faster spread and are harder impacted (i.e. [63]), rural areas have been focused less in the public debate and in research [21].

### Research needs and aims of the study

The literature review yielded several early study results regarding the effects of the measures to prevent the spread of the coronavirus as concerns traffic data. However, empirical findings related to the psychological backgrounds of the changes are rather thin on the ground because studies in behavioral science typically need long lead times. Yet, research is challenged to study how people adapted their mobility practices in terms of mode choice and the avoidance of trips as well as to estimate potential long-term effects of the virus era on transport mode choice, routines and working practices like giving priority to working from home. Furthermore, the study of psychological determinants and underlying mechanisms is relevant for predicting future behavior adaptation to change and for deriving measures to guide mobility behavior. A research gap opens up concerning the issues of equity, disparity and social justice, especially regarding the question which population groups are particularly affected by the indirect impacts of the crisis due to reduced personal mobility and isolation [17, 24, 57].

While traffic statistics and surveys in major cities point to an enormous reduction of personal mobility, especially for public transport modes [23, 36, 38], it is still unclear how transport demand changed in rural areas. Most of the existing studies are based on data from large metropolitan areas and global cities, such as New York [55]. However, the socio-structural and spatial characteristics of rural areas differ significantly from urban areas in terms of outward-bound commuter, average age, employment sector, number of cars, household size and distance to supply facilities and public utilities, among others. However, for a better understanding of travel demand and behavior during the pandemic situation and for deriving implications of the COVID-19 travel disruptions for longer-term changes in trip-making behavior it is essential to also gain knowledge about the reactions and thoughts of residents of rural areas. As empirical data is missing, it is unclear whether residents of disperse areas indeed perceive their surrounding as “safe shelters” ([21], p. 119) due to the living conditions that allow for social distancing better than urban areas.

Therefore, the current study aims at:

1. Providing subjective data concerning the changes of mobility practices and expected long-term effects of the coronavirus pandemic as perceived by affected persons, and
2. Extending the existing findings of urban settings by focusing on a rural area.

## Methods

To address the identified research gaps, a mixed-methods study was conducted that combined a qualitative interview study and a quantitative household survey. The integration of the two study types promises the benefit of connecting and embedding the data and consequently gaining additional insights [19]. The mixed-methods approach is seen as especially valuable in new research fields such, making it a suitable approach to the study of mobility changes due to the COVID-19 pandemic situation. Mixed methods can be defined as “research in which the investigator collects and analyses data, integrates the findings, and draws inferences using both qualitative and quantitative approaches or methods in a single study or a program of inquiry” ([56], p. 4). A key concept in this definition is the integration of data from different sources [56]. For this study, a qualitative interview study was conducted to gain insights into the mindset of persons related to their perception of the ongoing COVID-19 pandemic situation and its effect on their mobility behavior. The results informed the development of the questionnaire for the quantitative study. The rural district of *Altmarkkreis Salzwedel* in Northern Germany was chosen as a model case.

### Examined study area

The district of Altmarkkreis Salzwedel is located in the very North of the German federal state of Saxony-Anhalt. As of 2019, almost 85,000 people lived in the district, that covers an area of 2293 km^2^. With 38 inhabitants/km^2^, it is one of the districts with the lowest population density in Germany [27]. By June 2020, the district had a rather high share of unemployed persons with 7.1%, compared to Germany as a whole (6.2% [25];). With 7.0% the unemployment rate was nearly the same in June 2019 [25]. The purchasing power of Altmarkkreis Salzwedel residents was 44.064 € per household in 2018 and thus slightly higher than the mean for the state of Saxony-Anhalt (40.880 €, [8]).

### Concernment with the COVID-19 pandemic situation and containment measures taken in the study area

There were 34 proven COVID-19 infections until the end of July in the examined area [53]. With a share of 40.6 infected persons per 100.000 inhabitants, the district was one of the regions with a low infection rate [53]. In Germany, legal restrictions due to the corona pandemic, were mainly decided at federal state level. The German federal state of Saxony-Anhalt decided to shut down all community facilities, like schools and kindergartens and forbid all events with 50 or more participants as of March 16, 2020. At this point, Altmarkkreis Salzwedel had three proven COVID-19 cases. On March 22, the German government and German federal states agreed on further restrictions, especially for social contacts. Contacts with other people than members of one’s own household were to be minimized, and a minimum distance of 1.5 m was to be kept in public space [32]. Subsequently, on March 24, the state of Saxony-Anhalt announced that meetings with more than two persons were prohibited [26].

**Fig. 1:**
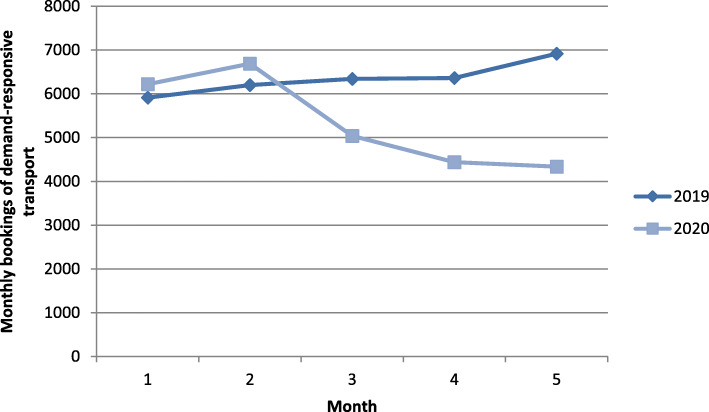
Comparison of bookings of demand-responsive transport in the first months of the years 2019 and 2020

The analysis of secondary data of the local public transport provider corroborates that residents’ transport demand decreased in the time period considered (May 2020). In comparison to the corresponding month of the previous year, bookings of the local demand-responsive bus system decreased to 79.4% in March, and 62.7% in May 2020 compared to 2019 (cf. Fig. 1). No statistics were available for other means of transport in the study area.

### Interview study

A telephone interview study with 15 residents was conducted by the authors themselves to gain in-depth insights into the changing mobility practices during the legal restrictions to stem the spread of the virus. The interviewees were contacted via publicly accessible phone books and randomly selected. For reaching the number of 15 interviews 45 numbers were called. The interview contained open questions related to the interviewees’ travel behavior and the changes due to the COVID-19 pandemic situation. It was structured by an interview guideline and took about 15 to 20 min. Of the interviewees, 8 were male and 7 female. The respondents’ age ranged from 11 to 85 years (*M* = 56.8 years, *SD* = 16.5 years). Two of them stated to be mobility-impaired. The interviews were conducted in April 2020 when legal restrictions to contain the spread of the virus were at their peak.

### Household survey

The findings of the qualitative study were enriched by a household survey study that was conducted in April and May 2020. The survey comprised questions regarding the shift of modal split, comparing the pre-pandemic status and the time of the corona pandemic, as well as the share of waived trips and the expected long-term changes of mobility practices. The questionnaire was distributed in 2.000 randomly selected households in the examined study area by direct mail. Furthermore, an online version of the questionnaire was used to reach more people. The online survey was disseminated via social media platforms. In total, 182 filled-out paper questionnaires were sent back (9.1% response rate) and 170 questionnaires were completed in the online survey. Out of the 170 online questionnaires, 52 were excluded because of more than 30% missing data, resulting 117 datasets for the analysis. However, the share of missing data, especially for sociodemographic data was rather high, as shown in Table 1. Data sets were included in cased where only individual questions lacked answers and there was no pattern in non-response shown. The mean age of respondents was 50.5 years (*SD* = 18.8 years) and thus slightly above the mean age of 47.4 years in the district of Altmarkkreis Salzwedel [10]. The number of unemployed respondents was lower (3.0%) than the unemployment rate in the district (7.1%, [31]). The share of respondents with a small household net income (< 25.000€/year or < 2.000€/year) was 31.9% and thus lower than for the district of Altmarkkreis Salzwedel (56%, [11]). Further sociodemographic characteristics of the study sample are summarized in Table 1.

**Table 1:** Sociodemographic characteristics of the study sample (*N* = 301)

Sociodemographic variable	Characteristics	n	%
Gender	Male	85	28.2
	Female	156	51.8
	Missing	60	20.0
Age	< 30 years	35	11.6
	30–44 years	51	16.9
	45–59 years	67	22.2
	> = 60 years	80	26.6
	Missing	68	22.6
Employment status	Full-time	88	29.2
	Part-time	41	13.6
	Unemployed	9	3.0
	Retired	57	18.9
	In education/training	29	9.6
	Temporarily out of work	3	1.0
	Home-maker	4	1.3
	Missing	70	23.3
Net household income	< 1.000 €	29	9.6
	1.000–2.000 €	67	22.3
	2.000–3.000 €	60	19.9
	3.000–4.000 €	36	12.0
	4.000–5.000 €	16	5.3
	> 5.000 €	6	2.0
	Missing	84	28.8
Driver’s license	Yes	267	88.7
	No	26	8.6
	Missing	8	2.6
Mobility impairments	Yes	23	7.7
	No	202	67.1
	Missing	60	20.0

### Data processing and analysis

The case study was analyzed with a mixed-methods approach combining qualitative content analysis of the interviews and statistical analysis of the survey results in a joint display [37]. The interviews were recorded and transcribed to be subsequently analyzed with the help of the software MAXQDA [59]. The survey data was analyzed using the statistical software SPSS [35]. The qualitative interviews were analyzed based on content analysis using an inductive approach [44]. The findings of both studies were analyzed in a mixed-methods approach using the four stages of the *Pillar Integration Process*: 1) listing, 2) matching, 3) checking and 4) pillar building [37].

## Results

### Qualitative findings

The analysis of the content of the interviews revealed two main clusters regarding the effects of the COVID-19 pandemic situation on the interviewees’ mobility behavior: 1) effects on distances travelled and number of trips, and 2) effects on transport mode choice.

Regarding the distances travelled and trips, two directions of changes were revealed in the analysis – a reduction of distances and number of trips (*n* = 16^1^), and unchanged distances and number of trips (*n* = 6). The majority of interviewees stated to have reduced their regular trips as a reaction to the COVID-19 pandemic situation, as illustrated by the quote: „*Well, I’m hardly mobile anymore because of the curfew. I have to stick to it. So for the moment I don’t need mobility.*“ (Interviewee 1, female, 58 years). Ceased trips were mainly daily commuting trips and trips with not strictly necessary purposes, such as leisure trips: „ *I am less mobile because I do not make trips for fun. Otherwise I always go somewhere for my hobby - photography. I have now made a reduction of 25 percent of my trips, I’d say.*” (Interviewee 10, male, 58 years). However, there are also other interview partners that stated not to have changed their mobility practices since the outbreak of the crisis: *“Since I work in the village, everything has actually remained the same.*“ (Interviewee 6, female, 41 years). Others even expressed their resistance to change their mobility behavior and showed lower commitment to preventive behavior: “*Nothing has changed for me. Because the bottom line is, from my point of view, there’s nothing you can do about a virus. What are you going to do about a virus? You can only drive yourself crazy, but when it’s your turn, it’s your turn.*” (Interviewee 2, male, 65 years).

Regarding transport mode choice, the analysis revealed two tendencies in responses – a change (*n* = 5) and no change of behavior (*n* = 7). In more detail, a change in the transport modes was mostly reported for trips by car. Interview partners stated to have reduced trips by car due to the fact that daily commuting trips by car were no longer necessary: “*So my car’s basically parked in the yard for two weeks.*” (Interviewee 14, male, 40 years). Furthermore, the same interview partner expressed the reduction of trips by train and plane as an effect of canceled business trips. Although four interview partners stated to have used the bus regularly before the pandemic situation, the interviews revealed no changes in public bus usage since it remained equally seldom as before: “*It [bus trips] was also rare before.* “(Interviewee 3, female, 40 years). None of the interviewees expressed strong rejection to use the public bus or fear related to the risk of getting infected while using public transport. The possibility of using the bus without the need to pay for a ticket due to the closing of the driver’s cab was not seen as an incentive to use the bus more often: „*But I must say that even though it [rides by bus] is free now, I still don’t go more than absolutely necessary. You would think that I go to Salzwedel every day just because I don’t have to pay. But no, I do not do that either.* “(Interviewee 7, female, 60 years).

Besides the changes of mobility behavior as a reaction to the COVID-19 pandemic situation, the interviews revealed some interesting insights into the mindset of the interviewees, concerning some perceived positive side effects of the situation. A father of a family stated to notice the positive aspects of his working from home – “*So for me, who usually drives an hour to work, it’s a distance. Now I’ve gained two hours, if you like. So, in principle, I spend more time with the kids. So a bit of luck in misfortune*” (Interviewee 11, male, 38 years). Another interview partner referred to the solidarity of her fellow citizens “*At the moment, in these Corona times, we have neighbourhood meetings here. And they’re on the phone a couple times a week expecting any requests. And I take advantage of that. Yesterday, for example, they did some shopping for me. And, yes, I’m using that anyway. I don’t know if other people use it much, but I use it.* “(Interviewee 4, female, 85 years).

### Quantitative findings

The aim of the household survey was to quantify the changes in mobility behavior as a reaction to the CODID-19 pandemic situation. The following section is divided into two subsections 1) changes in mobility behavior and 2) modal shift. The results are reported descriptively and with the help of statistical analyses (e.g. variance analysis and regression analysis for describing interdependencies).

#### Changes in mobility behavior

The changes in respondents’ mobility behavior were measured by self-assessments on items regarding the potential change of mobility behavior “*Have you changed your mobility behavior (e.g. choice of means of transport and distances travelled) due to the current situation caused by the coronavirus? [yes / no] If yes, please give reasons for your answer”* and the share of reduced trips “*How many of your usual trips are you currently foregoing due to the political guidelines for behavior in the corona crisis? Please estimate how many trips you are currently foregoing in percent*”. The analysis revealed that 30.2% of respondents reported a change in mobility behavior as a reaction to the COVID-19 pandemic situation, whereas 50.5% did not perceive a change (19.3% missing because of skipped question). Thus, out of all valid responses, slightly more than one third of the respondents (*n* = 91, 37.4%) stated to have changed their mobility and nearly two thirds did not (*n* = 152, 62.6%). When asked for the share of trips they reduced as a reaction to the crisis, the range of responses was very wide from 0 to 100%. In more detail, 34 respondents (16.0%) stated not to have reduced their trips at all. The mean share of omitted trips was considerable with 33.49% (*SD* = 27.5%). The share of persons that stated to have changed their mobility behavior was higher for retired persons (46.9%) than for employed persons (33.3%) whereas the mean share of reduced trips was nearly the same for both groups (retired: 34.6%, *SD* = 27.8%, employed: 32.26%, *SD* = 27.64%).

Further analyses revealed a connection between the possession of a driving licence and the perceived mobility changes due to the COVID-19 pandemic situation in a way that persons without a driving licence reported more often to have changed their mobility. This relationship was marginally significant in a *Chi*^*2*^-test (χ^2^(2) = 4.915, *p* = .086, *n* = 243). Female gender was linked to a significantly higher share of reduced trips (*M* = 37.6%, *SD* = 27.9%) as a consequence of the pandemic situation than male gender (*M* = 26.7%, *SD* = 25.8%, *t(*202) = 2.799, *p* = .006). The effect size of *r* = .193 was small.

The respondents were asked to give further explanations for their experienced changes. The majority of the explanations referred to the introduction of remote work (*n* = 18) and the cancelation of classes (*n* = 5). Some explanations also referred to the reduction of trips for pleasure and leisure activities that caused a reduction of mobility (*n* = 13). Further explanations concerned the ban of inter-state travel during the previous months (*n* = 7).

#### Modal shift

Modal split in terms of frequency of transport mode use was measured using a Likert scale (*1 = (nearly) daily, 2 = 3–6 times/week, 3 = 1–2 times/week, 4 = 1–3 times/month, 5 = never)*. Reported frequency of use before vs. during the coronavirus pandemic situation was compared for each mode, using *Wilcoxon signed-rank test,* due to the non-parametric data structure. The analysis revealed several significant changes for the use of different transport modes (cf. Table 2). A significant reduction of trips was shown for all transport means (see below) except trips by foot (*z* = − 1.042, *p* = .297), by motorcycle (*z* = − 1.732, *p* = .083) and by demand-responsive transport (*z* = − 1.656, *p* = .098). These modes were apparently not significantly affected by the pandemic situation. During the pandemic situation, the private car was used significantly less than before the pandemic situation (z = − 2.697, *p* = .007)). However, the effect was small (*r* = .162). A strong reduction was also shown for the public transport means of bus (*z* = − 4.205, *p* < .001, *r* = .253) and train (*z* = − 3.620, *p* < .001, *r* = .217). For rides by bike, a significant, but small increase was revealed (*z* = − 3.010, *p* = .003, *r* = .181). For bus transport and motorcycle the share of respondents who nearly never used them during pre-corona time was very high (*n* = 181 for bus, *n* = 209 for motorcycle). Thus, when looking only at the sub-sample of persons who had used the public bus service before on a regular basis, the statistical analysis revealed a significant difference between the two point in time (*z* = − 5.035, *p* < .001, *r* = .610), in a way that bus transport was chosen less often than before the crisis. No difference was shown for the sub-sample of motorcycle users (*z* = − 1.134, *p* = .257).

**Table 2:** Comparison of modal split before and during the corona pandemic situation (*N* = 275)

Means of transport	Point in time	Median	Modal shift	*z*	*p*	r
Car	before	1.00	−0.120	−2.697	.007*	.162
	during	2.00				
Bike	before	3.00	0.147	−3.010	.003*	.181
	during	3.00				
Bus	before	5.00	−0.195	−4.205	<.001**	.253
	during	5.00				
Train	before	5.00	−0.122	−3.620	<.001**	.217
	during	5.00				
By foot	before	2.00	−0.064	−1.042	.297	.063
	during	2.00				
Motorcycle	before	5.00	0.026	− 1.732	.083	.104
	during	5.00				
DRT	before	5.00	−0.046	−1.656	.098	.099
	during	5.00				

* = *p* < .05, ** = *p* < .01, *DRT* demand-responsive transport, *1 = (nearly) daily* to *5 = never*

The analysis of modal shift revealed that the reduction of trips by car was significantly related to a higher net household income (*r* = −.164, *p* = .018). No significant correlation was shown for age and a change of number of trips by car or any other means of transport However, employed persons report a higher reduction in car usage than retired persons (*U* = 2649.5, *p* = .007), however, their frequency of car usage was higher before (Mdn = 1.00) than the one of retired persons (Mdn = 3.00). No differences in modal shift were shown for the comparison of female and male gender.

**Fig. 2:**
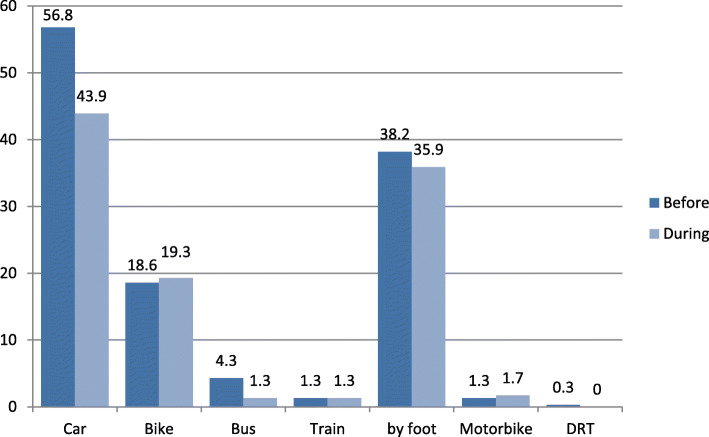
Share of daily usage according to means of transport

Figure 2 shows the change inf daily usage of different means of transport as a consequence of the COVID-19 pandemic situation. As shown here, especially the share of respondents that use the private car daily was reduced from 56.8% to 43.9%, resulting in a reduction to 77. % of the value before the crisis. This change was even more substantial for public bus usage. The share of respondents that stated to have used the bus before on a daily basis was reduced to 23.3% of the value before the spread of the virus, while the initial share was already low. A reduction was also reported in trips by foot (from 38.2% to 35.9%). The reported frequency of trips by bike and motorbike remained rather constant, with a small tendency of increase (from 18.6% to 19.3%, and 1.3% to 1.7%, respectively).

#### Perceived long-term effects

**Fig. 3:**
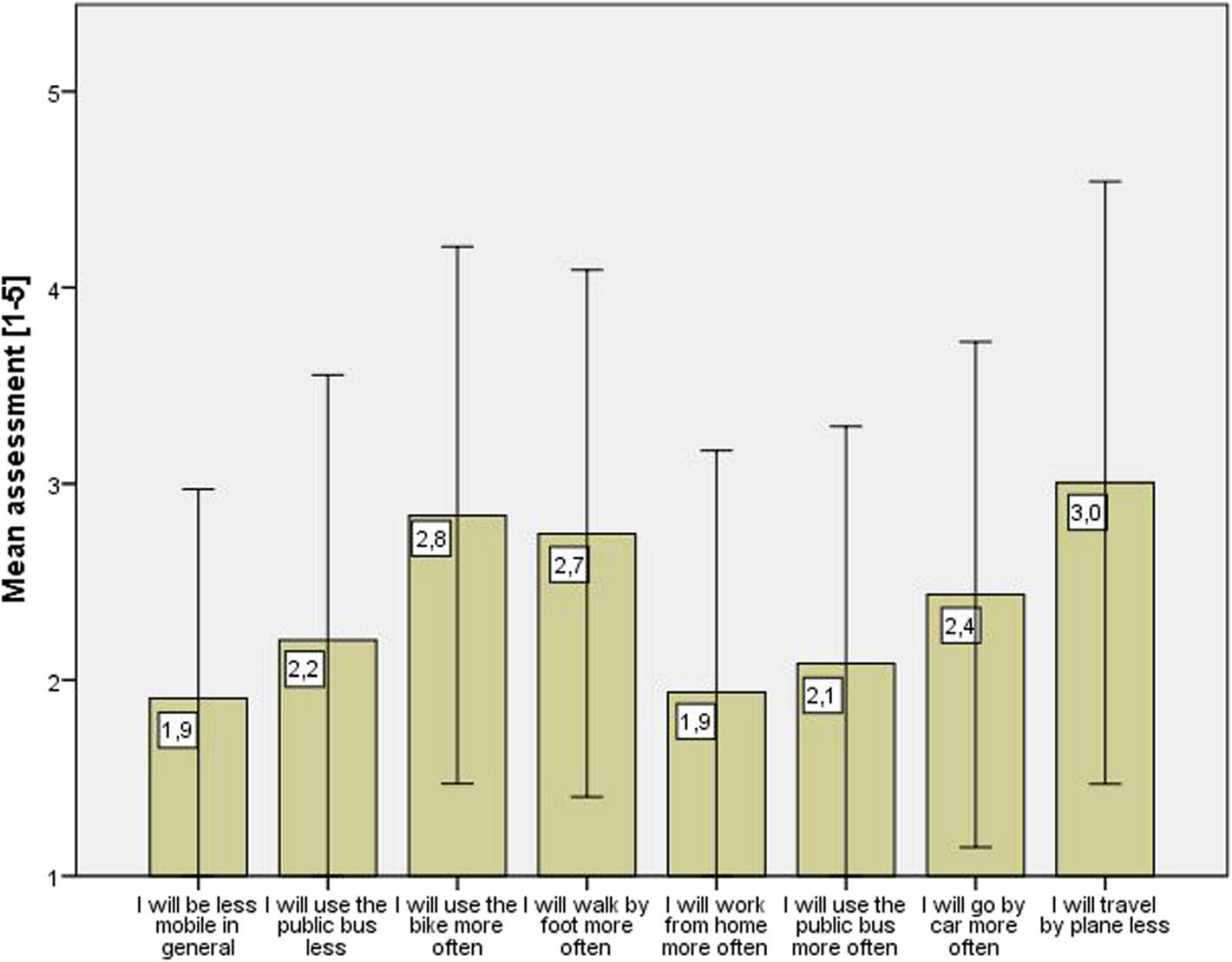
Mean assessment of long-term changes in mode choice. *Note*. Whiskers represent 1 standard deviation. 1 = fully disagree, 5 = fully agree

The perceived long-term effects of the COVID-19 pandemic situation on the respondents’ mobility behavior were assessed with the question “*Do you think that your mobility behavior will change in the long term (in the coming months and years) as a result of the current pandemic situation?*”, using a Likert scale from *1 – fully disagree* to 5 *= fully agree* to answer to the proposed statements (e.g. *I will use the public bus less*). Overall, the respondents assessed the long-term effects of the situation on their mobility behavior to be weak, as shown by the mean values that only exceed the value of 3 for the item *“I will travel by plane less”*. Yet, the high standard deviations, illustrated by the whiskers in Fig. 3, point to a strong variation of respondents’ assessments. Respondents do rather not expect the likelihood as low that they will be less mobile in general in the future (*M* = 1.96, *SD* = 1.11), as shown in Fig. 3. The same applies for the assessment of the likelihood of more remote work. Employed respondents did mostly not agree that they will work from home more often in the future (*M* = 2.06, *SD* = 1.30). Respondents partially agreed that they will use bikes more often (*M* = 2.94, *SD* = 1.36) and travel less by plane (*M* = 3.04, *SD* = 1.55). The two contrary questions concerning bus usage (*“I will use the public bus less”* and *“I will use the public bus more often”*), reveal that respondents neither think that they will reduce their trips by bus nor that they will increase trips by bus in the future.

Regarding the effect of sociodemographic characteristics on the perceived long-term effects, the employment status had a significant effect on the assessment of the item *“I will be less mobile in general”*. Retired persons agreed to this statement more (*M* = 2.44, *SD* = 1.285) than employed persons (*M* = 1.87, *SD* = 1.04, *U =* 1867.5, *p* = .009). There was no significant tendency for female respondents to assess long-term effects on a general mobility reduction as slightly more likely (*M* = 2.06, *SD* = 1.17) than male respondents (*M* = 1.77, *SD* = 0.98, *U* = 4720.0, *p* = .092). Respondents that stated to have used bus transport less than once a week before the pandemic expressed less agreement to the statement that they will use public bus more often in the future (*M* = 1.96, *SD* = 1.11) than respondents that have used the bus more often (*M* = 2.85, *SD* = 1.46, *U* = 1416.0, *p* = .002).

**Fig. 4:**
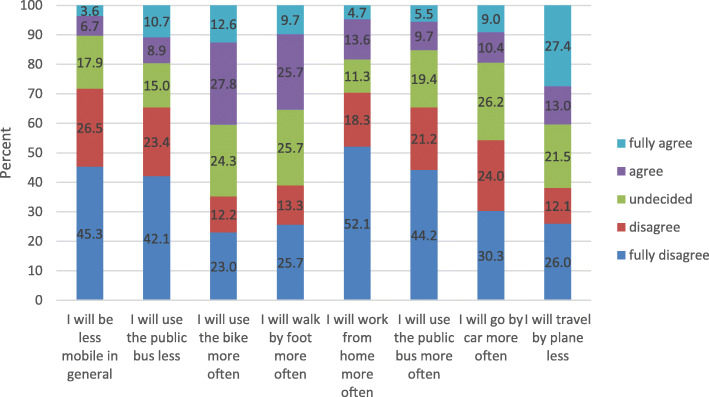
Assessment of long-term changes according displayed by frequency of options (*N* = 226, rest missing)

Figure 4 presents a further way of analysing the respondents’ assessment of long-term effects. As shown here, the frequency of agreement to the statements differs strongly. Whereas more than 40% of respondents agreed or fully agreed that they will use the bike more often in the future as a reaction to the corona crisis situation, the agreement to the statement to be less mobile in general was weaker (10.3% agreed or fully agreed). A high agreement was found for the statements regarding a reduction of flights (40.3% agreed or fully agreed) and for an increase in trips by foot (35.4% agreed or fully agreed). More than half of the respondents fully disagreed that they will work from home more often.

### Mixed findings

The findings of both studies were integrated and embedded with the help of a joint display according to the Pillar Integration Process [37]. For this purpose, findings of the qualitative and quantitative study were compared and contrasted (c.f. Table 3). The pillar themes column in the center contains the integrated inferences about the patterns that emerged from the comparison of the studies.

**Table 3:**
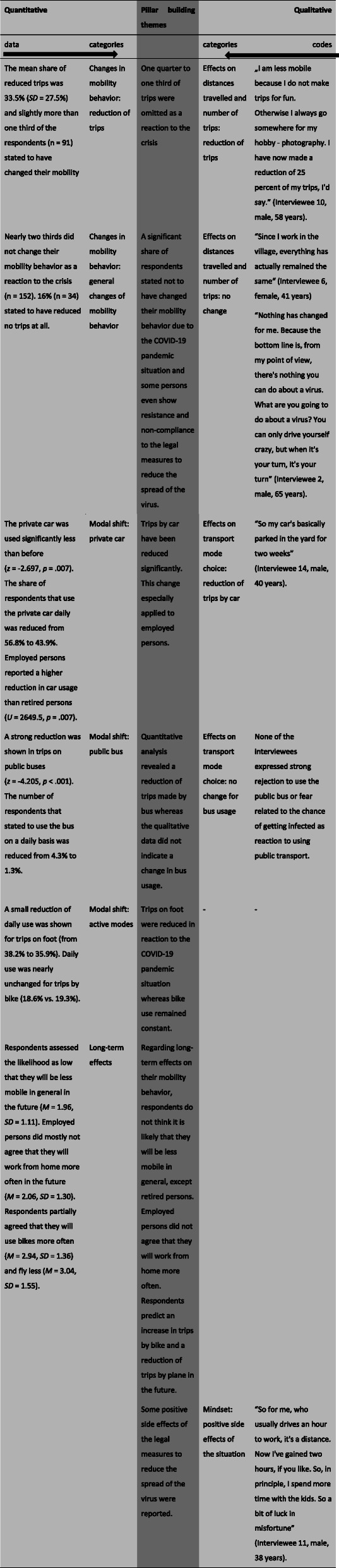
Joint Display of qualitative and quantitative findings

## Discussion

### Methodological considerations and limitations of the study

Before interpreting and discussing the results, we would like to reflect on the method and discuss some limitations. A limitation results from the rather high item nonresponse for some of the sociodemographic characteristics, such as gender or income. Nonresponse on such items may be explained by perceptions that the questions are too sensitive or personal [49] or certain beliefs about the survey, for example that a question is not relevant for the topic of the survey [54]. Thus, a potential for bias due to the nonresponse should be considered in the interpretation of the results [22]. The same holds for the (probably) unequal distribution of gender in the survey sample. However, a weight adjustment did not change the statistical results and was therefore not applied to the data, in consideration of the possible drawbacks, such as additional biases and increased variance [30]. Thus, to sum up, the specific characteristics of the sample should be considered for interpretation of the results.

Furthermore, the findings on the two questions regarding mobility changes as a reaction to the corona virus pandemic should be further reflected, as they seem to contradict each other: Respondents reported reduced trips, but did not perceive a change of their mobility behavior. One possible explanation for this may be the order of the questions, with the abstract assessment of potential changes preceding the specific report of behavior. Presumably, if the specific question regarding the share of reduced trips had been asked first, this could have led participants to also reflect the changes in their general assessment.

### Interpretation and assessment of the findings

The aim of the mixed-methods analysis was to approach the rising research need to study mobility changes in reaction to the global COVID-19 pandemic situation. In more detail, the study focused on two research needs: 1) To provide subjective data concerning the changes of mobility practices and expected long-term effects as perceived by affected persons and 2) to broaden the existing findings from urban settings by focusing on a rural area.

Regarding the first research aim, the findings provide several insights into the perception of changes in mobility behavior. The findings imply that not nearly every respondent changed his or her mobility practices compared to the pre-pandemic status. As the merging of the qualitative and quantitative study showed, there is a considerable share of respondents that stated to have not changed their mobility behavior nor reduced trips as a reaction to the COVID-19 pandemic situation. In more detail, 16% stated to have reduced no trips at all and the mean value of reduced trips was rather low with 33.5%. These findings are in contrast to the findings from further studies that revealed an enormous reduction of personal mobility in cities [36, 38, 52]. One explanation is based on the finding that reductions in travel are mainly caused by the reduction of everyday commute [36]. However, the share of residents of the examined study area that are employed in the primary (5.5%) and secondary sector (29.9%) is higher compared to a city like Berlin (0.0% and 13.6% respectively in 2018, [9]). These structural characteristics might impede remote work. Accordingly, the share of persons that stated to have changed their mobility behavior was lower for employed persons than for retired persons. This finding is in accordance with the result that employed respondents believe that that they will not work from home more often in the future. These findings imply that even though some persons that would have liked to change their mobility might have not because of external conditions like the impossibility of remote working or using food delivery services in rural areas.

Interestingly, the quantitative analysis revealed a clear reduction of trips by bus compared to the time before the corona crisis situation whereas interview partners in the qualitative study did not express a change in the number of trips by bus. This might be explained by the fact that the four interviewed persons that uses bus transport regularly are captive riders as they do not own a driver’s licence or access to a car.

The comparison of the modal split before and during the corona virus era revealed a modal shift away from public transport means and motorized individual transport towards the intensified use of bicycles. In contrast to studies from urban areas [36], the share of trips by foot did not increase compared to the pre-pandemic situation, which might be explained by the spatial characteristics of rural areas that entail longer distances. However, the share of trips by foot before the corona crisis was small. Under consideration of a reduction trip by other means of transport, like bus, the modal share of walking increased somehow. The findings of an increase of car use in modal split in recent studies [4, 23, 34] could not be replicated in this study. In contrast, the respondents report a reduction of trips by car on the modal share and a reduction of daily use, which was also found in a study by de Haas, Faber and Hamersma [20]. A possible explanation might be the relatively high share of car trips on modal split in rural areas [28]. Thus, based on a so-called *ceiling effect* [18], a reduction of trips by car is more likely than a further increase. Giving regard to the second research goal to broaden the existing findings of urban settings on rural areas, the study shows that insights that were found for urban settings cannot be simply transferred to rural areas. The challenge of transferring findings has been shown by Li, Rudolph & Mennis [40] who found urban–rural differences in the relationship between reductions in population mobility and the growth rate in COVID-19 cases. From a policy perspective the research points out that the same regulations might result in different outcomes regarding mobility behavior in different places. Thus, policy should be cautious in the use of broad travel bans but search for specific and adapted regulations. Whereas cities implemented temporary pop-up cycle lanes [50] or car-free city centers [15] similar approaches for rural areas are missing. Adding on this, policy is asked to provide more accessibility to services to residents of rural areas to increase livability of disperse areas as an alternative to progressing urbanization [16]. Furthermore, the results suggest that a reduction of trips is not always as possible in rural areas as in urban areas where jobs that allow for remote working are more common. Thus, when having a possible next pandemic situation in mind, policy is challenged to ensure digital connections for facilitating remote working in rural areas.

The matched data enhanced our understanding of the mechanisms that were operating within the context. The merging of the qualitative and quantitative results provides some explanatory approaches for the findings. For example, the relatively low number of respondents that stated to have changed their mobility behavior as a reaction to the crisis could be explained by the statements of the interviews that indicate some respondents’ perception of no need for changes or even resistance to change and non-compliance.

Concerning the perceived long-term effects of the COVID-19 virus era, the majority of respondents assume the effects of the current situation on their long-term mobility behavior to be weak. Respondents assessed the likelihood as low that they will be less mobile in general in the future. However, retired persons agree more frequently with the statement that they will be less mobile in the future. This finding might be related to the general reduction of mobility for increasing age and the fact that the COVID-19 pandemic represents a major threat particularly to older adults [46]. In accordance to a study by de Haas, Faber and Hamersma [20], respondents partially agreed that they will use bikes more often in the future. Furthermore, a considerable number of respondents expected that the COVID-19 pandemic situation will result in a long-term reduction of trips by plane which was also found in studies before [20, 36]. In line with the findings of a study from the Netherlands [20], a considerable part of respondents does not expect to work from home more often in the future. Budd and Ison [14] propose a new concept of *Responsible Transport* in recognition of the pandemic situation and for post-COVID recovery. Responsible Transport requires an element of individual responsibility that involves the decision whether traveling is really necessary or can be avoided and the impact of travel choices on others. It will be shown in the future, whether today’s predictions that were stated by the respondents will be translated into action. Giving regard to the point in time when the survey was conducted (April and May 2020), the study presents insights into the mind of persons at the beginning of the pandemic situation. At this point of time, the assessment of changed mobility habits as a reaction to the coronavirus situation was to early. However, behavior change often co-occurs with important events in people’s lives (i.e. [42]) and events, like a pandemic have the power to disrupt long-existing and stable habits according to the *Habit Discontinuity Hypothesis* [60]. Thus, further behavioral research, like panel studies are needed to study the effects of the crisis of changes of mobility habits, like the priority position of the car in rural areas.

### Further research needs

As other recent publications, this study represents an approach to address the new research issues that are rising as an effect of the novel COVID-19 situation. Several research questions still remain open that call for further analyses. One emerging issue is the need to study human needs in isolation, especially of elderly and persons living alone that might be of particular relevance in disperse areas [7, 43]. Furthermore, the perception of long-term effects presents an interesting starting point for further research. Studies could focus on the effects on mode choice and the perception of risks when using public transport. Adding onto this, the construct *perceived behavioral control* of attitude-based mobility behavior models needs further investigations in the light of changing mobility behavior since the pandemic situation shifts mobility choices from internal to more external control. Giving regard to the fact, that it is still too early to investigate the stability of changes in mobility behavior, research is requested to assess the effects of the crisis and the measures to prevent it on mobility habits.

## Conclusions

The study provides insights into the effects of the COVID-19 era on the mobility behavior of a rural case study sample in Germany. Thereby, the study assesses the immediate impacts of the measures imposed to prevent the spread of the virus on the movement of people in a defined area. Using a mixed-methods design, the study provides insights into the perception and mindset of affected persons regarding the changes of mobility practices due to the measures to prevent the spread of the virus. The results reveal a modal shift towards the reduction of trips by car and bus and an increase of trips by bike. In contrast findings from major cities, no increase in trips by foot occurred due to the corona pandemic situation [36]. The findings indicate that the study participants do not experience strong cuts in their mobility and do not predict strong long-term effects on their general mobility.

## Data Availability

The datasets supporting the conclusions of this article are included in additional files.

## Abbreviations

COVID-19: Coronavirus disease
DRT: Demand-responsive transport
N: Number of respondents
SD: Standard deviation
U: Value of the Mann-Whitney-*U*-Test

## Footnotes

1 The number of codes does not represent the number of participants since more than one expression of a person can be counted.

## References

[1] Abouk, R., & Heydari, B. (2020). The immediate effect of covid-19 policies on social distancing behavior in the united states. Available at: https://www.medrxiv.org/content/medrxiv/early/2020/04/28/2020.04.07.20057356.full.pdf 10.1177/0033354920976575 PMC8093844 33400622

[2] ADAC (2020). Corona und Mobilität: Mehr Homeoffice, weniger Berufsverkehr. Available online: https://www.adac.de/verkehr/standpunkte-studien/mobilitaets-trends/corona-mobilitaet/

[3] Ajzen, I. (1991). The theory of planned behavior. *Organizational Behavior and Human Decision Processes*, *50*(2), 179–211. 10.1016/0749-5978(91)90020-T.

[4] Aloi, A., Alonso, B., Benavente, J., et al. (2020). Effects of the COVID-19 lockdown on urban mobility: empirical evidence from the city of Santander (Spain). *Sustainability*, *12*(9), 3870.

[5] Apple (2020). Mobility Trends Report. Available online: https://www.apple.com/covid19/mobility (Accessed 24 June 2020).

[6] Arellana, J., Márquez, L., & Cantillo, V. (2020). COVID-19 outbreak in Colombia: An analysis of its impacts on transport systems. *Journal of Advanced Transportation*, *2020*. 10.1155/2020/8867316.

[7] Armitage, R., & Nellums, L. B. (2020). COVID-19 and the consequences of isolating the elderly. *The Lancet Public Health*, *5*(5), e256. 32199471 10.1016/S2468-2667(20)30061-X PMC7104160

[8] Bertelsmann Stiftung (2020a). Statistische Daten. Qualifikation. Wegweiser-Kommune.de. Available online: https://www.wegweiser-kommune.de/statistik/altmarkkreis-salzwedel-lk+qualifikation+2016-2018+tabelle (Accessed 22 Jul 2020).

[9] Bertelsmann Stiftung (2020b). Statistische Daten. Wirtschaft & Arbeit – Beschäftigung. Wegweiser-Kommune.de. Available online: https://www.wegweiser-kommune.de/statistik/altmarkkreis-salzwedel-lk+beschaeftigung+2016-2018+berlin+tabelle (Accessed 25 Jul 2020)

[10] Bertelsmann Stiftung (2020c). Demographiebericht. Ein Baustein des Wegweisers Kommune. Altmarkkreis Salzwedel. Available online: https://www.wegweiser-kommune.de/kommunale-berichte/demographiebericht

[11] Bertelsmann Stiftung (2020d). Statistische Daten – Soziale Lage. Available online: https://www.wegweiser-kommune.de/statistik/altmarkkreis-salzwedel-lk+soziale-lage+2016-2018+tabelle

[12] Bish, A., & Michie, S. (2010). Demographic and attitudinal determinants of protective behaviours during a pandemic: A review. *British Journal of Health Psychology*, *15*(Pt 4), 797–824. 10.1348/135910710X485826. 20109274 10.1348/135910710X485826 PMC7185452

[13] Bucsky, P. (2020). Modal share changes due to COVID-19: The case of Budapest. *Transportation Research Interdisciplinary Perspectives*, *100141*. 10.1016/j.trip.2020.100141. 10.1016/j.trip.2020.100141 PMC7290209 34171021

[14] Budd, L., & Ison, S. (2020). Responsible transport: A post-COVID agenda for transport policy and practice. *Transportation Research Interdisciplinary Perspectives*, *6*, 100151. 10.1016/j.trip.2020.100151. 34173454 10.1016/j.trip.2020.100151 PMC7311912

[15] Connolly, K. (2020). 'Cleaner and greener': Covid-19 prompts world's cities to free public space of cars. The Guardian, 18^th^ May 2020. Online available: https://www.theguardian.com/world/2020/may/18/cleaner-and-greener-covid-19-prompts-worlds-cities-to-free-public-space-of-cars

[16] Cotella, G., & Vitale Brovarone, E. (2020). Questioning urbanisation models in the face of Covid-19. *Tema. Journal of Land Use, Mobility and Environment*, 105–118. 10.6092/1970-9870/6869.

[17] Coven, J., & Gupta, A. (2020). Disparities in mobility responses to COVID-19. NYU Stern Working Paper.

[18] Cramer, D., & Howitt, D. L. (2004). *The sage dictionary of statistics: A practical resource for students in the social sciences*. Thousand Oaks: Sage.

[19] Creswell, J. W., & Plano Clark, V. L. (2007). *Designing and conducting mixed methods research*. Thousand Oaks: Sage.

[20] de Haas, M., Faber, R., & Hamersma, M. (2020). How COVID-19 and the Dutch ‘intelligent lockdown’change activities, work and travel behaviour: Evidence from longitudinal data in the Netherlands. *Transportation Research Interdisciplinary Perspectives*, *100150*. 10.1016/j.trip.2020.100150 PMC7284275 34171019

[21] de Luca, C., Tondelli, S., & Åberg, H. (2020). The Covid-19 pandemic effects in rural areas. *TEMA Journal of Land Use, Mobility and Environment*, 119–132. 10.6092/1970-9870/6844.

[22] Dong, Y., & Peng, C. Y. J. (2013). Principled missing data methods for researchers. *SpringerPlus*, *2*(1), 222. 23853744 10.1186/2193-1801-2-222 PMC3701793

[23] Eisenmann, C., Kolarova, V., Nobis, C., Winkler, C. & Lenz, B. (2020). DLR-Befragung: Wie verändert Corona unsere Mobilität? Available online: https://verkehrsforschung.dlr.de/de/news/dlr-befragung-wie-veraendert-corona-unsere-mobilitaet (Accessed 24 June 2020)

[24] Engle, S., Stromme, J., & Zhou, A. (2020). Staying at home: Mobility effects of covid-19. Working paper. Retrieved from: http://johnstromme.com/research/Engle_Stromme_Zhou_COVID_WP.pdf

[25] Federal Employment Agency (2020). Labour market at a glance - Reporting month June 2020 - Altmarkkreis Salzwedel. Available online: https://statistik.arbeitsagentur.de/Navigation/Statistik/Statistik-nach-Regionen/Politische-Gebietsstruktur/Sachsen-Anhalt/Altmarkkreis-Salzwedel-Nav.html (Accessed 22 Jul 2020).

[26] Federal Government of Saxony-Anhalt (2020). Zweite Verordnung über Maßnahmen zur Eindämmung der Ausbreitung des neuartigen Coronavirus SARS-CoV-2 in Sachsen-Anhalt (Zweite SARS-CoV-2-Eindämmungsverordnung-2.SARS-CoV-2-EindV). Legal regulation. Available online: https://www.lkjl.de/media/dokumente/corona/vo_zweite_sars-co-2-eindaemmungsvo_final.pdf (Accessed 22 Jul 2020).

[27] Federal Ministry of Food and Agriculture (2016). Bevölkerungsdichte. Homepage oft he Federal Ministry of Food and Agriculture. Retrieved online: https://www.landatlas.de/laendlich/bevdichte.html (Accessed 22 Jul 2020).

[28] Follmer, R., & Gruschwitz, D. (2019). *Mobility in Germany – Short report. Edition 4.0 of the study by infas, DLR, IVT and infas 360 on behalf of the Federal Ministry of transport and digital Inftrastructure (BMVI), Bonn*.

[29] Gao, S., Rao, J., Kang, Y., Liang, Y., & Kruse, J. (2020). Mapping county-level mobility pattern changes in the United States in response to COVID-19. *SIGSPATIAL Special*, *12*(1), 16–26.

[30] Gelman, A. (2007). Struggles with survey weighting and regression modeling. *Statistical Science*, *22*(2), 153–164.

[31] German Federal Employment Agency (2020). Arbeitsmarkt im Überblick - Berichtsmonat Oktober 2020 - Altmarkkreis Salzwedel. Online available: https://statistik.arbeitsagentur.de/Auswahl/raeumlicher-Geltungsbereich/Politische-Gebietsstruktur/Kreise/Sachsen-Anhalt/15081-Altmarkkreis-Salzwedel.html

[32] German Government (2020). Besprechung von Bundeskanzlerin Merkel mit den Regierungschefinnen und Regierungschefs der Länder zum Coronavirus. Press release. 22.03.2020. Available online: https://www.bundesregierung.de/breg-de/themen/coronavirus/besprechung-von-bundeskanzlerin-merkel-mit-den-regierungschefinnen-und-regierungschefs-der-laender-zum-coronavirus-1733266 (Accessed 22 Jul 2020).

[33] Google (2020). See how your community is moving around differently due to COVID-19. Available online: https://www.google.com/covid19/mobility/?hl=en-GB (Accessed 24 June 2020).

[34] Huang, J., Wang, H., Fan, M., Zhuo, A., Sun, Y., & Li, Y. (2020). Understanding the impact of the COVID-19 pandemic on transportation-related behaviors with human mobility data. In *Proceedings of the 26th ACM SIGKDD International Conference on Knowledge Discovery & Data Mining*, (pp. 3443–3450).

[35] IBM Analytics (2018). SPSS [software]. Available online: https://www.ibm.com/analytics/de/de/technology/spss/

[36] Infas (2020). Alles anders oder nicht? Unsere Alltagsmobilität in der Zeit von Ausgangsbeschränkung oder Quarantäne. Retrieved online: https://www.infas.de/fileadmin/user_upload/infas_mobility_CoronaTracking_Nr.03_20200513.pdf

[37] Johnson, R. E., Grove, A. L., & Clarke, A. (2019). Pillar integration process: A joint display technique to integrate data in mixed methods research. *Journal of Mixed Methods Research*, *13*(3), 301–320.

[38] Klein, B., LaRocky, T., McCabey, S., Torresy, L., Privitera, F., Lake, B., ... Scarpino, S. V. (2020). Assessing changes in commuting and individual mobility in major metropolitan areas in the United States during the COVID-19 outbreak. Retrieved online: https://uploads-ssl.webflow.com/5c9104426f6f88ac129ef3d2/5e8374ee75221201609ab586_Assessing_mobility_changes_in_the_United_States_during_the_COVID_19_outbreak.pdf

[39] Kraemer, M. U., Yang, C. H., Gutierrez, B., Wu, C. H., Klein, B., Pigott, D. M., … Brownstein, J. S. (2020). The effect of human mobility and control measures on the COVID-19 epidemic in China. *Science*, *368*(6490), 493–497. 32213647 10.1126/science.abb4218 PMC7146642

[40] Li, X., Rudolph, A. E., & Mennis, J. (2020). Association Between Population Mobility Reductions and New COVID-19 Diagnoses in the United States Along the Urban–Rural Gradient, February–April, 2020. *Preventing Chronic Disease*, *17*:200241. 10.5888/pcd17.200241 PMC7553217 33006542

[41] Marsden, G., Anable, J., Chatterton, T., Docherty, I., Faulconbridge, J., Murray, L., … Shires, J. (2020). Studying disruptive events: Innovations in behaviour, opportunities for lower carbon transport policy? *Transport Policy*, *94*, 89–101. 10.1016/j.tranpol.2020.04.008.

[42] Marsden, G., & Docherty, I. (2013). Insights on disruptions as opportunities for transport policy change. *Transportation Research Part A*, *51*, 46–55. 10.1016/j.tra.2016.07.006.

[43] Matias, T., Dominski, F. H., & Marks, D. F. (2020). Human needs in COVID-19 isolation. *Journal of Health Psychology*, *25*(7). 10.1177/1359105320925149. 10.1177/1359105320925149 32375564

[44] Mayring, P. (2004). Qualitative content analysis. *A Companion to Qualitative Research*, *1*(2004), 159–176.

[45] Molloy, J, C. Tchervenkov, B. Hintermann, K.W. Axhausen (2020). Tracing the Sars-CoV-2 impact: The first month in Switzerland, Arbeitsberichte Verkehrs- und Raumplanung, 1503, IVT, ETH Zürich, Zürich

[46] Morley, J. E., & Vellas, B. (2020). COVID-19 and older adult. *The Journal of Nutrition, Health & Aging*, *24*(4), 364–365. 10.1007/s12603-020-1349-9. 10.1007/s12603-020-1349-9 PMC7113379 32242202

[47] National Academies of Sciences, Engineering, and Medicine (2020a). Research needs statements specific to transportation and pandemics. Available online: http://www.trb.org/main/CallforRNSTransportationAndPandemics.aspx (last Accessed: 24 June 2020)

[48] National Academies of Sciences, Engineering, and Medicine (2020b). *Rapid expert consultation on social distancing for the COVID-19 pandemic (March 19, 2020)*. Washington, DC: The National Academies Press. 10.17226/25753.

[49] National Research Council (2002). Adjusting for Missing Data in Low-Income Surveys. In *Studies of Welfare Populations: Data Collection and Research Issues*, (pp. 129–156). Washington, DC: The National Academies Press. 10.17226/10206.

[50] Panozzo, N., & Kabell, M. (2020). ECF recommendations for healthier and safer streets after the coronavirus pandemic. Discussion paper. Online available: https://repository.difu.de/jspui/bitstream/difu/576720/1/Recommendations_for_post_COVID_cities.pdf

[51] Pepe, E., Bajardi, P., Gauvin, L., Privitera, F., Lake, B., Cattuto, C., & Tizzoni, M. (2020). COVID-19 outbreak response, a dataset to assess mobility changes in Italy following national lockdown. *Scientific Data*, *7*(1), 1–7. 32641758 10.1038/s41597-020-00575-2 PMC7343837

[52] Pullano, G., Valdano, E., Scarpa, N., Rubrichi, S., & Colizza, V. (2020). *Population mobility reductions during COVID-19 epidemic in France under lockdown*. 10.1101/2020.05.29.20097097. 10.1016/S2589-7500(20)30243-0 PMC7598368 33163951

[53] Robert Koch-Institut (2020). COVID-19-Dashboard. Available online: https://experience.arcgis.com/experience/478220a4c454480e823b17327b2bf1d4 (last Accessed 22 Jul 2020).

[54] Rogelberg, S. G., Fisher, G. G., Maynard, D. C., Hakel, M. D., & Horvath, M. (2001). Attitudes toward surveys: Development of a measure and its relationship to respondent behavior. *Organizational Research Methods*, *4*(1), 3–25.

[55] Sirkeci, I., & Yucesahin, M. M. (2020). Coronavirus and migration: Analysis of human mobility and the spread of COVID-19. *Migration Letters*, *17*(2), 379–398.

[56] Tashakkori, A., & Creswell, J. W. (2007). The new era of mixed methods. *Journal of Mixed Methods Research*, *1*, 3–7. 10.1177/2345678906293042.

[57] van Dorn, A., Cooney, R. E., & Sabin, M. L. (2020). COVID-19 exacerbating inequalities in the US. *Lancet (London, England)*, *395*(10232), 1243. 32305087 10.1016/S0140-6736(20)30893-X PMC7162639

[58] Venkatesh, V., Morris, M. G., Davis, G. B., & Davis, F. D. (2003). User acceptance of information technology: Toward a unified view. *MIS Quarterly*, *27*(3), 425–478.

[59] VERBI GmbH (2020). *MAXQDA, Software für qualitative Datenanalyse, 1989–2019, VERBI Software*. Berlin: Consult. Sozialforschung GmbH.

[60] Verplanken, B., Roy, D., & Whitmarsh, L. (2018). Cracks in the wall: Habit discontinuities as vehicles for behaviour change. In *The Psychology of habit*, (pp. 189–205). Cham: Springer. 10.1007/978-3-319-97529-0_11.

[61] Warren, M. S., & Skillman, S. W. (2020). Mobility changes in response to COVID-19. arXiv preprint arXiv:2003.14228.

[62] World Health Organization (2020). Recommendations to Member States to improve hand hygiene practices to help prevent the transmission of the COVID-19 virus. https://www.who.int/publications/i/item/recommendations-to-member-states-to-improve-hand-hygiene-practices-to-help-prevent-the-transmission-of-the-covid-19-virus.

[63] Wu, X., Nethery, R. C., Sabath, M. B., Braun, D., & Dominici, F. (2020). Exposure to air pollution and COVID-19 mortality in the United States: A nationwide cross-sectional study. *MedRxiv preprint*. 10.1017/CBO9781107415324.004.

[64] Yildirim, M., Geçer, E., & Akgül, Ö. (2020). The impacts of vulnerability, perceived risk, and fear on preventive behaviours against COVID-19. *Psychology, Health & Medicine*, *26*(1), 35-43 10.1080/13548506.2020.1776891 32490689

